# Cancer Immunology with a Focus on Understudied Cancers as Targets for Immunotherapy

**DOI:** 10.3390/ijms18010127

**Published:** 2017-01-11

**Authors:** M. Rita I. Young

**Affiliations:** 1 Research Service (151), Ralph H. Johnson Veterans Affairs Medical Center, 109 Bee Street, Charleston, SC 29401, USA; rita.young@va.gov; Tel.: +1-843-792-9953; 2 Department of Otolaryngology—Head and Neck Surgery, Medical University of South Carolina, 173 Ashley Avenue, Charleston, SC 29425, USA

A number of immune therapeutic approaches have been transitioning from being experimental to being incorporated as standard approaches, either alone or in conjunction with other therapies. However, this has not been uniformly implemented or even tested across all cancer types. For example, immune therapies ranging from checkpoint inhibitory antibodies, cytokine therapies and tumor vaccines have been extensively tested and used as a treatment for melanoma. Immunotherapy for lung cancers has also received considerable attention. While immunotherapeutic approaches to treatment of leukemia have become quite common, this is been more sporadic in solid tumors. Since immunotherapy has not been uniformly implemented or even tested across solid cancer types, this Editorial and the articles in this special issue focused on immunotherapy for solid cancers and, in particular, solid cancers that have been understudied as targets for immunotherapy.

It is important to note that there are differences in how the terms “extensively tested” or “understudied” are determined. A PubMed review of over 1000 citations pertaining to cancer and immunotherapy showed a significantly greater number of original published works related to immunotherapy for melanoma and lung cancer than for other cancer types (Figure 1). However, a review of over 500 clinical trials in ClinicalTrials.gov involving immunological interventions for cancer patients revealed lung cancer and, instead of melanoma, breast cancer to be the most common cancers being targeted (Figure 2). While clinical trials involving immunological interventions for patients with pancreatic and ovarian cancers appeared at a high frequency in ClinicalTrials.gov (Available online: https://clinicaltrials.gov/), published results involving such trials were more rare compared to results studies involving of immunotherapies for patients with other cancer types.

The focus of this special edition has been on cancers that have received more limited attention as targets for immunotherapy or for which new immunological approaches have lagged or been stagnant. Below (Table 1) is a summary of the original research articles that represent this Special Issue entitled “Cancer Immunology with a Focus on Understudied Cancers as Targets for Immunotherapy” in *International Journal of Molecular Sciences*.

In addition to these original research papers, the Special Issue also contains a number of review papers exploring new immunological treatment targets [8] and treatment approaches for lung cancer [9], multiple myeloma [10], ovarian cancer [11] and colorectal cancer [12].

It is recognized cancers should not all be lumped into a single category as they express different antigens and establish microenvironments that differ, in part based on the cancer location and the immune modulators that they express. The challenges of immunotherapy have been a topic of interest and have recently extensive reviews in the literature [13,14,15,16,17,18]. Some exhibit more profound levels of immune suppression than others, thereby increasing the challenge of immunotherapeutic strategies. Others show more inflammatory phenotypes. However, lessons learned from immunological treatments for one cancer type can be used to help guide treatment approaches for other cancer types. It is hoped that immunological treatments that have been tested and shown to be effective for cancers that have been more frequently studied can be tested in cancers that have less frequently been considered for immunotherapy. It is also hoped that the studies described in this special edition can readily wind their way from diagnostic approaches to identification of new target antigens, in vitro feasibility analyses, in vivo studies in orthotopic animal models and, finally, to cancer patients.

## Figures and Tables

**Figure 1:**
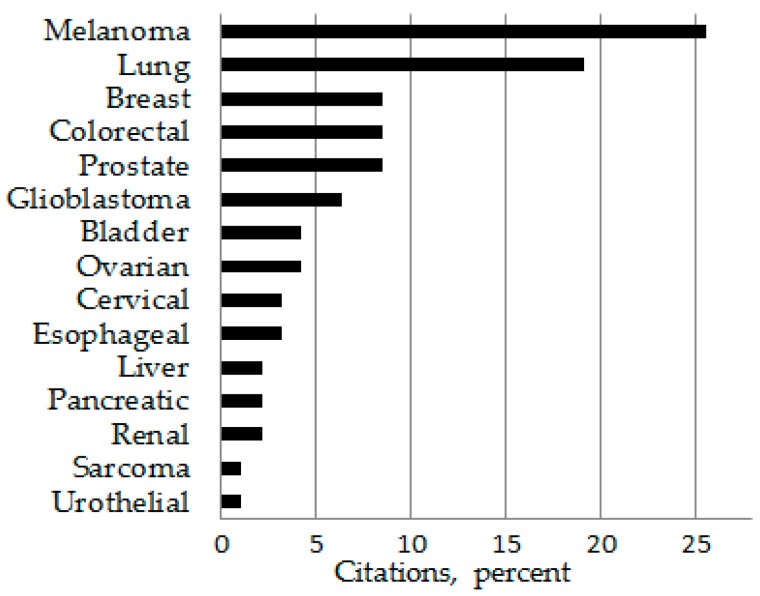
Cancer immunotherapy citations in PubMed.

**Figure 2:**
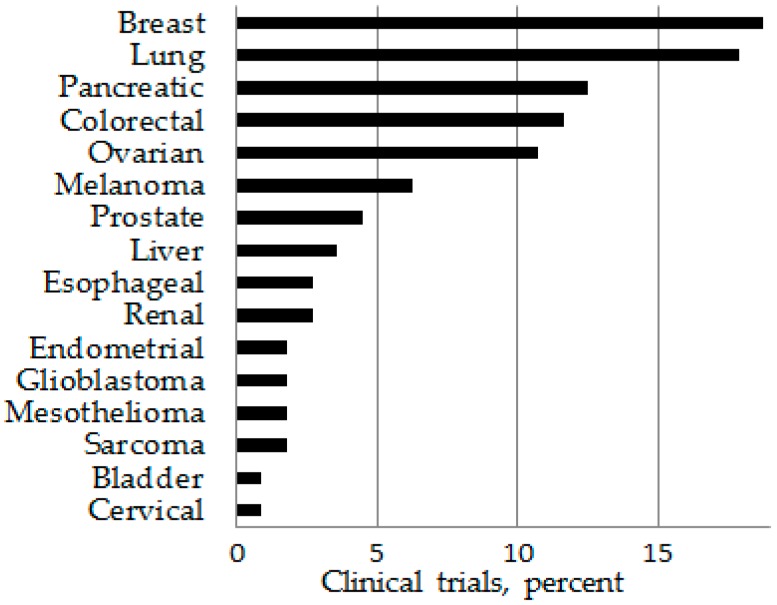
Immunotherapy trials in ClinicalTrial.gov.

**Table 1:** Summary of original research articles in Special Issue “Cancer Immunology with a Focus on Understudied Cancers as Targets for Immunotherapy”.

Topic	Cancer Type	Focus	References
Targets and biomarkers	Uterine cancer	MUC1 and hTERT as T-cell targets	[1]
	Primary brain cancers	Nuclear factor erythroid 2-related factor as a biomarker for cancer grade	[2]
In vitro: Feasibility of treatment	Lymphoma	Combination of brentuximab vedotin (antibody-drug conjugate) plus cytokine-induced killer cells	[3]
In vivo: Treatment strategy	Brain glioma	Semi-allogeneic tumor vaccine	[4]
	Bladder cancer	Intravesical papillomavirus non-replicative pseudovirions carrying thymidine kinase gene combined with Ganciclovir treatment	[5]
	Pancreatic ductal adenocarcinoma	HIF-1 to recruit inflammatory macrophages via CCL2	[6]
	Breast cancer	Engineered anti-HER-2 A21 chimeric antibody	[7]
